# Supplementary material for: Regulatory effects of the Uty/Ddx3y locus on neighboring chromosome Y genes and autosomal mRNA transcripts in adult mouse non-reproductive cells
Source: Sci Rep. 2020 Sep 10;10:14900. doi: 10.1038/s41598-020-71447-3 (PMC7484786; doi:10.1038/s41598-020-71447-3)
Supplement: Supplementary file 1 — Supplementary Information. [file 41598_2020_71447_MOESM1_ESM.docx]

**SUPPLEMENTARY FILES**

**Regulatory effects of the *Uty*/*Ddx3y* locus on neighboring chromosome Y genes and autosomal mRNA transcripts in adult mouse non-reproductive cells**

Christian F. Deschepper

Cardiovascular Biology Research Unit, Institut de recherches cliniques de Montréal (IRCM) and Université de Montréal

**Supplementary tables:**

**Supplementary Table S1:** List of mouse genes with highest levels of co-expression with Uty, ranked on the basis of mutual ranking (MR) scores

**Supplementary Table S2:** Top most differentially accessible regions in chromatin from cardiomyocyte nuclei from either WT of *Uty^GT^* mice, as determined by ATAC-Seq assays. Regions formatted in bold correspond to those identified in the context of other experiments. Regions with FDR significance < E-09 are shown only if they are flanked by other regions with some levels of significance.

**Supplementary Table S3:** List of human genes with highest levels of co-expression with Uty, as determined on the basis of mutual ranking (MR) scores. Genes are shown in shaded cells when other neighboring genes also show evidence of interaction with UTY.

**Supplemental Table S4:** Coordinates of genomic regions predicted to be transcriptionally silent in cardiomyocytes. The negligeable FPKM values obtained for these regions in samples from either Y*^UtyGT^* mice or WT mice (identified as either Uty* or b6*) were interpreted as evidence for the absence of DNA contaminationin the nuclear RNA samples.

**Supplementary Table S5:** List of oligomer primers used for RT-qPCR amplification

**Supplementary Table S6** is provided in an additional file as a four tab-containing Excel spreadsheet. Each sheet lists all GO terms showing both high (> 2 fold) and significant (FDR < 0.01) enrichment among genes affected by *Uty^GT^* (either down- or upregulated) in either left ventricles (LV) or cardiomyocyocytes (CM).

**Supplementary Table S7** is provided in an additional file as a four tab-containing Excel spreadsheet. It contains information about genes showing either up- or down-regulation in left ventricles from *Uty^GT^* mice, as well as a more comprehensive list of GO categories showing significant enrichment for these genes.

**Supplementary Table S8** is provided in an additional file as a four tab-containing Excel spreadsheet. It contains information about genes showing either up- or down-regulation in cardiomyocytes from *Uty^GT^* mice, as well as a more comprehensive list of GO categories showing significant enrichment for these genes.

**Supplementary Table S9** is provided in an additional file as a three tab-containing Excel spreadsheet. The first tab shows a Venn diagram for genes affected by *Uty^GT^* in mouse cardiac left ventricles vs. those affected by *UTY* knockdown in human macrophages; the second tab shows the identity of the 31 genes showing overlap; the third tab shows the GO term enrichment analysis within the group of 31 overlapping genes.

**Supplementary figures:**

**Supplementary Fig. S1:** **RT-qPCR quantification of abundance of mRNA transcripts of *Utx, Ddx3x and Eif2s3x in left ventricular extracts from Uty^GT^ and WT mice.*** Expression of *Utx* was unaffected, *Ddx3x* showed a slight increase of about 10 % (**P* < 0.05), expression of *Eif2s3x* was increased by about 40% (*P* < 0.01).

**Supplementary Fig. S2:** Localization of loci embedded within introns of either Uty or Ddx3y and containing sequences corresponding to putatively expressed transcripts.

**Supplemental Fig S3**: **Graphic representation of ATAC-Seq results for chr17.** Regions shown contain peaks showing significant differences in intensity between *Uty^GT^* and WT mice. For comparison, ENCODE tracks obtained using extracts from adult mouse hearts for either H3K27ac, p300 or Pol2 ChIP-Seq or Dnase hypersensitivity assays are also shown (in red). Loci of known genes are shown in the top part of the figure (in blue).

**Supplemental Fig S4**: Full images of the hybridized membranes that have been used for the top part of Fig. 2 in the manuscript.

**Supplemental Fig S5**: Full images of the two half hybridized membranes that have been hybridized with either anti-Ddx3 or anti-GAPDH antibodies, and used for the bottom part of Fig. 2 in the manuscript.

**Supplementary Table S1: List of mouse genes with highest levels of co-expression with Uty, ranked on the basis of mutual ranking (MR) scores**

| **Rank** | **Gene** | **Chr** | **Gene type** | **Entrez Gene ID** | **MR** |
| --- | --- | --- | --- | --- | --- |
| 0 | Uty | chrY:1,097,144-1,245,718 | protein coding | 22290 | 0 |
| 1 | Ddx3y | chrY:1,260,771-1,286,629 | protein coding | 26900 | 2.13 |
| 2 | Eif2s3y | chrY:1,010,543-1,028,594 | protein coding | 26908 | 2.26 |
| 3 | Kdm5d | chrY:897,790-946,316 | protein coding | 20592 | 2.28 |
| 4 | Gm39552 | chrY:1,114,067-1,120,066 | lncRNA | 105243746 | 25.03 |
| 5 | C030026M15Rik | chrY:1,161,073-1,162,805 | lncRNA | 77378 | 116.33 |
| 6 | Uba1y | chrY:2,264,257-2,278,687 | protein coding | 22202 | 218.38 |
| 7 | Gm35612 | chrX:50,537,389-50,562,598 | lncRNA | 102639259 | 750.5 |
| 8 | Usp9y | chrY:1,298,961-1,459,782 | protein coding | 107868 | 1590.67 |
| 9 | 4932431L22Rik | chrY:70,367,444-70,370,583 | lncRNA | 619557 | 2289.96 |

Co-expression values are expressed as “mutual ranking scores”, which corresponded to the mean geometric mean of the correlation rank of gene A to gene B and of gene B to gene A. Calculations were performed with tools provided by COEXPRESdb, using data from 2236 Affymetrix arrays (microarray platform GPL1261) performed on multiple tissues across 154 experiments. A detailed list of all tissues used is available at the following link: (<https://coxpresdb.jp/static/help/GPL1261v2_samples.shtml>). The MR scores are inversely proportional to the strength of co-expression levels (the lower the score, the stronger the interaction). MR scores higher than 200 are considered as weak.

**Supplementary Table S2: Top most differentially accessible regions in chromatin from cardiomyocyte nuclei from either WT of *Uty^GT^* mice, as determined by ATAC-Seq assays.**

| **Gene Name** | **Chr** | **Start** | **End** |  | **FDR** |
| --- | --- | --- | --- | --- | --- |
| **Uty** | **chrY** | **581836** | **582382** | **Promoter-TSS** | **3.50E-34** |
| En2 | chr5 | 28495275 | 28496134 | First intron | 1.02E-31 |
| **Ddx3y** | **chrY** | **622332** | **623154** | **First intron** | **2.86E-18** |
| **Eif2s3y** | **chrY** | **346529** | **347077** | **Promoter-TSS** | **5.50E-14** |
| **Malat1** | **chr19** | **5821944** | **5823927** | **Intergenic** | **9.64E-19** |
| Neat1 | chr19 | 5824319 | 5825012 | TTS | 8.61E-13 |
| Neat1 | chr19 | 5831054 | 5832841 | Non-coding | 2.23E-09 |
| Neat1 | chr19 | 5838441 | 5842512 | Non-coding | 8.52E-09 |
| Neat1 | chr19 | 5828977 | 5829566 | Non-coding | 1.12E-08 |
| Neat1 | chr19 | 5829981 | 5830325 | Non-coding | 3.04E-07 |
| Neat1 | chr19 | 5837107 | 5838143 | Non-coding | 1.43E-06 |
| Neat1 | chr19 | 5835340 | 5836037 | Non-coding | 2.01E-06 |
| Neat1 | chr19 | 5833417 | 5833811 | Non-coding | 2.73E-04 |
|  |  |  |  |  |  |
| Myh7 | chr14 | 55619384 | 55620431 | Promoter-TSS | 1.60E-09 |
| Myh7 | chr14 | 55620990 | 55624433 | Intergenic | 1.16E-06 |
| Myh7 | chr14 | 55612681 | 55615300 | Intergenic | 2.74E-06 |
| Myh7 | chr14 | 55608473 | 55609053 | Intron 12 of 40 | 4.91E-05 |
| Myh7 | chr14 | 55615784 | 55616173 | Intergenic | 1.78E-03 |
| Myh7 | chr14 | 55606934 | 55608000 | Intron 15 of 40 | 1.86E-02 |
|  |  |  |  |  |  |
| Zbtb16 | chr9 | 48632529 | 48633073 |  | 5.69E-09 |
| Zbtb16 | chr9 | 48574572 | 48575021 |  | 1.91E-05 |
| Zbtb16 | chr9 | 48564539 | 48564746 |  | 2.63E-04 |
| Zbtb16 | chr9 | 48590823 | 48591848 |  | 4.93E-04 |
| Zbtb16 | chr9 | 48605695 | 48605938 |  | 7.61E-04 |
| Zbtb16 | chr9 | 48568245 | 48568945 |  | 2.21E-02 |
| Zbtb16 | chr9 | 48636958 | 48637403 |  | 2.73E-02 |
| Zbtb16 | chr9 | 48622674 | 48623269 |  | 4.43E-02 |
|  |  |  |  |  |  |
| Plin4 | chr17 | 56242595 | 56246147 |  | 2.13E-08 |
| Plin4 | chr17 | 56239471 | 56241031 |  | 1.88E-06 |

**Supplementary Table S3: List of human genes with highest levels of co-expression with Uty,as determined on the basis of mutual ranking (MR) scores**

| **Rank** | **Symbol** | **chr** | **Gene type** | **Entrez Gene ID** | **MR** |
| --- | --- | --- | --- | --- | --- |
| **0** | **UTY** | **chrY:13,248,379-13,480,673** | **protein coding** | **7404** | **0** |
| **5** | **DDX3Y** | **chrY:12,904,868-12,920,478** | **protein coding** | **8653** | **16.89** |
| **1** | **USP9Y** | **chrY:12,662,368-12,860,802** | **protein coding** | **8287** | **7.45** |
| 6 | TTTY15 | chrY:12,662,367-12,692,224 | lncRNA | 64595 | 23.11 |
|  |  |  |  |  |  |
| **2** | **KDM5D** | **chrY:19,705,417-19,744,939** | **protein coding** | **8284** | **9.64** |
| 3 | TXLNGY | chrY:19,588,609-19,593,602 | pseudo-gene | 246126 | 11.07 |
| 17 | LOC107987345 | chrY: 19,039,939 – 19,049,347 | pseudogene | 107987345 | 388.97 |
| 18 | BCORP1 | chrY:19,456,394-19,567,092 | pseudogene | 286554 | 431.15 |
| 15 | LOC105377223 | chrY:19,552,934-19,563,950 | lncRNA | 105377223 | 342.69 |
|  |  |  |  |  |  |
| **4** | **ZFY** | **chrY:2,966,844-3,002,626** | **protein coding** | **7544** | **12.12** |
| 21 | ZFY-AS1 | chrY:2,966,844-3,002,626 | lncRNA | 100506003 | 828.15 |
|  |  |  |  |  |  |
| **7** | **EIF1AY** | **chrY:20,575,776-20,593,154** | **protein coding** | **9086** | **39.88** |
| 11 | TTTY10 | chrY:20,464,916-20,575,497 | lncRNA | 246119 | 105.32 |
| 20 | LOC107987347 | chrY:21,240,255-21,302,707 | lncRNA | 107987347 | 711.98 |
| 22 | LOC105377225 | chrY:21,138,363-21,170,221 | lncRNA | 105377225 | 1037.03 |
|  |  |  |  |  |  |
| **8** | **PRKY** | **chrY:7,274,313-7,371,868** | **protein coding** | **5616** | **75.99** |
| **9** | **CYorf15B** | **chr1:32,179,675-32,198,285** | **protein coding** | **84663** | **84.83** |
| **12** | **NLGN4Y** | **chrY:14,523,967-14,622,220** | **protein coding** | **22829** | **106.44** |
| 13 | TMSB4Y | chrY:13,703,899-13,706,024 | lncRNA | 9087 | 120.78 |
| 14 | LINC00278 | chrY:3,002,894-3,120,705 | lncRNA | 100873962 | 255.24 |
| 16 | NCRNA00185 | chrY:18,872,501-18,878,228 | lncRNA | 55410 | 343.25 |
| 24 | FAM224A | chrY:18,326,253-18,353,210 | lncRNA | 401630 | 3018.95 |
| 19 | TBL1Y | chrY:6,910,686-7,091,683 | lncRNA | 90665 | 608.41 |
| 23 | PCDH11Y | chrY:5,056,090-5,104,700 | lncRNA | 83259 | 2602.03 |

**Supplemental Table S4**

| **ensembl_gene_id** | **Coordinates mm10** | **length (bp)** | **b6_11** | **b6_15** | **b6_31** | **uty_11** | **uty_15** | **uty_31** |
| --- | --- | --- | --- | --- | --- | --- | --- | --- |
| ENSMUSG00000008789 | chr7:17,713,252-17,761,121 | 47,870 bp | 0.00 | 0.00 | 0.00 | 0.00 | 0.00 | 0.00 |
| ENSMUSG00000019785 | chr10:33,512,334-33,624,600 | 112,267 bp | 0.00 | 0.00 | 0.00 | 0.00 | 0.00 | 0.00 |
| ENSMUSG00000020633 | chr12:28,437,795-28,548,337 | 110,543 bp | 0.00 | 0.00 | 0.00 | 0.00 | 0.00 | 0.00 |
| ENSMUSG00000000560 | chr5:70,961,057-71,095,849 | 134,793 bp | 0.04 | 0.07 | 0.13 | 0.10 | 0.11 | 0.06 |
| ENSMUSG00000001260 | chr5:70,751,047-70,842,617 | 91,571 bp | 0.00 | 0.00 | 0.00 | 0.00 | 0.00 | 0.00 |
| ENSMUSG00000018589 | chrX:165,129,017-165,326,981 | 197,965 bp | 0.00 | 0.00 | 0.00 | 0.00 | 0.00 | 0.00 |
| ENSMUSG00000022112 | chr14:115,092,215-116,525,192 | 1,432,978 bp | 0.00 | 0.00 | 0.00 | 0.00 | 0.00 | 0.00 |
| ENSMUSG00000017688 | chr3:3,508,030-3,659,800 | 151,771 bp | 0.00 | 0.00 | 0.00 | 0.00 | 0.00 | 0.00 |
| ENSMUSG00000022076 | chr14:96,105,265-96,519,034 | 413,770 bp | 0.00 | 0.00 | 0.00 | 0.00 | 0.00 | 0.00 |
| ENSMUSG00000009356 | chr11:87,806,428-87,826,114 | 19,687 bp. | 0.00 | 0.00 | 0.00 | 0.00 | 0.00 | 0.00 |
| ENSMUSG00000021363 | chr13:41,025,120-41,079,706 | 54,587 bp | 0.00 | 0.00 | 0.00 | 0.00 | 0.00 | 0.01 |
| ENSMUSG00000005493 | chr3:153,857,141-153,906,138 | 48,998 bp | 0.18 | 0.10 | 0.15 | 0.22 | 0.10 | 0.22 |
| ENSMUSG00000010505 | chr2:181,763,332-181,827,775 | 64,444 bp | 0.00 | 0.00 | 0.00 | 0.00 | 0.00 | 0.00 |
| ENSMUSG00000004231 | chr19:44,757,394-44,837,871 | 80,478 bp | 0.00 | 0.00 | 0.00 | 0.00 | 0.01 | 0.00 |
| ENSMUSG00000021852 | chr14:49,298,520-49,525,837 | 227,318 bp | 0.00 | 0.00 | 0.00 | 0.01 | 0.01 | 0.00 |
| ENSMUSG00000010435 | chr8:12,573,049-12,600,738 | 27,690 bp | 0.00 | 0.00 | 0.00 | 0.00 | 0.00 | 0.00 |
| ENSMUSG00000003271 | chr7:45,729,983-45,759,555 | 29,573 bp | 0.00 | 0.01 | 0.00 | 0.00 | 0.00 | 0.00 |
| ENSMUSG00000001670 | chr8:109,990,436-109,999,804 | 9,369 bp | 0.00 | 0.00 | 0.00 | 0.00 | 0.00 | 0.00 |
| ENSMUSG00000021541 | chr13:56,773,098-56,895,789 | 122,692 bp | 0.00 | 0.00 | 0.00 | 0.00 | 0.00 | 0.03 |

**Supplementary Table S5: List of oligomer primers used for RT-qPCR amplification**

| **Gene** | **Forward** | **Reverse** |
| --- | --- | --- |
| *Rps16* | GCTACCAGGGCCTTTGAGATG | AGGAGCGATTTGCTGGTGTGG |
| *Uty* | ATGGAGAAGGGCATGAGAAC | AGTTGGTGGTCTTGGAGGTG |
| *Ddx3y* | GTGCCTTCTTGGTTGGAAAG | AATCCAGCATTTGCAGAACC |
| *Eif2s3y* | AGGAGGCAGAGTTAGTGCAG | CTTCTTATCTGGCCCCAACC |
| *Utx* | TGAAAGTTCCAGGAAGCAGAA | TTCAAAACACCCCAGTAGCC |
| *Ddx3x* | GGAAGATATGGCCGTCGTAAA | GACGGACTCTAGATCGGTATGA |
| *Eif2s3x* | GGGAGGTGTAGCTGGTGGTA | GTTCCGCAAAAAGTGACACA |

**Supplementary Fig. S1:**

**Expression of Utx, Ddx3x and Eif2s3x in left ventricles from *Uty^GT^* and WT mice**

**Supplementary Fig S2:**

**Supplementary Fig S3:**

**
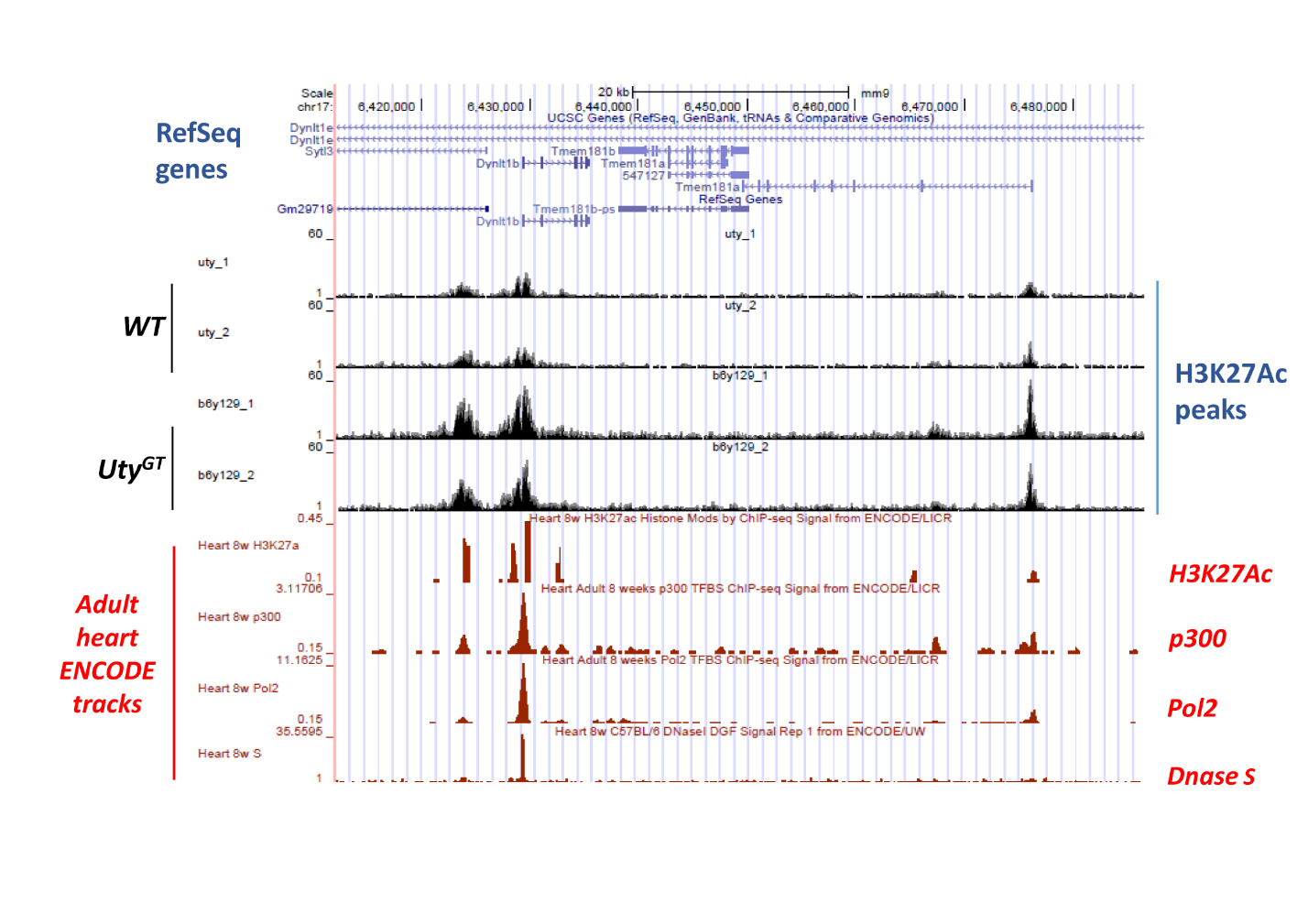
**

**Supplemental Fig. S4:**

**Supplemental Fig. S5**
